# Surveillance after treatment for head and neck cancer

**DOI:** 10.1097/MOO.0000000000001102

**Published:** 2026-01-12

**Authors:** Petri Koivunen

**Affiliations:** Oulu University Hospital, Otorhinolaryngology and Head and Neck Surgery, University of Oulu, Clinical Medicine Research Unit, Oulu, Finland

**Keywords:** head and neck cancer, imaging, recurrence, surveillance

## Abstract

**Purpose of review:**

Systematic follow-up protocols, often including regular imaging, are an essential component of posttreatment care for head and neck cancer, aimed at the early detection of disease relapse and mitigating treatment-related morbidity. However, there is no consensus on the optimal length of follow-up and the value of imaging in surveillance.

**Recent findings:**

Most head and neck cancer recurrences occur within 1–2 years after treatment. After 2 years, the recurrence rate decreases significantly, and after 3 years, recurrences are infrequent. Most of the recent studies suggest that prolonged scheduled follow-up programs are not necessary, as asymptomatic salvageable late recurrences are rare. Imaging surveillance for the early detection of otherwise undetected recurrences is supported by many studies, although its impact on survival remains unclear.

**Summary:**

Recent literature emphasizes intensive follow-up programs during 1–2 years, as well as patient education for self-observation of alarming symptoms. Prolonged surveillance programs after 2–3 years may not be effective in detecting asymptomatic recurrences. While personalized surveillance based on risk factors is suggested, a lack of strict evidence hampers stratification. Imaging may be of value in detecting early asymptomatic recurrences at least up to 2 years, but disagreement exists regarding its utility in improving survival.

## INTRODUCTION

Posttreatment follow-up (FU) is universally adopted as part of the treatment protocol for head and neck cancer (HNC) patients, and it is generally regarded as indispensable for controlling side effects and detecting recurrences [1–3]. Since about 50% of patients with advanced disease experience recurrences, the important rationale for most patients’ adherence to follow-up programs is to detect possible recurrences as early as possible to still enable curative-intent treatment.

The correlation between intensive follow-up and survival or disease control seems to be lacking [1,4,5]. Challenges with resources, concerns about cost-efficacy and the value of intensive and prolonged follow-up programs in improving recurrence detection and survival rate have risen discussion for more individualized and focused follow-up programs.

There is no general agreement about the optimal follow-up schedule, and the guidelines do have large variation of recommended scheduled visit intervals, necessity of radiological imaging and length of surveillance. In general, the recommendation for assessment during first year varies between 1 and 3 months and during second year 2–5 months. Length of recommended follow-up varies between 3 and 10 years [6–9]. The value of imaging in surveillance for detecting recurrences is also controversial [10]. 

**Box 1:**
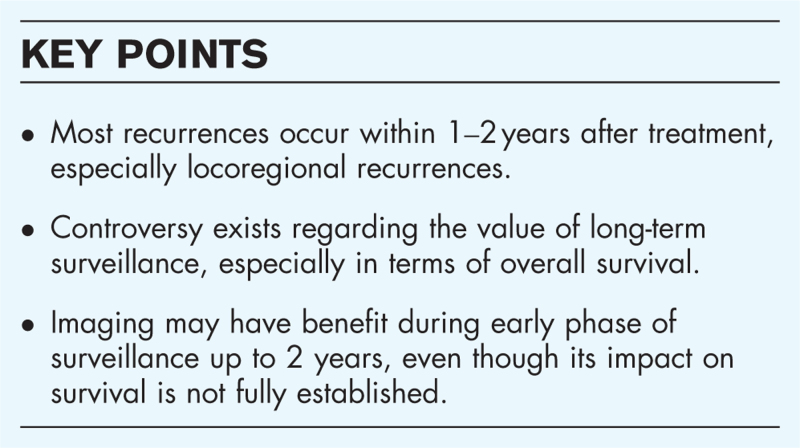
no caption available

## EFFICACY OF FOLLOW-UP

### Time frame of recurrences

A rational approach to assessing the optimal intensity and length of a follow-up program is to evaluate the time frame in which most recurrences occur. According to some older studies including HNC from various sites, most of the locoregional recurrences are detected during 1 year after treatment and 76 and 95% of cases are detected until second year after treatment [11].

Detailed analysis of recurrences was done in the large nationwide study, which evaluated the time frame of recurrences, second primary tumours, distant metastases and survival of 1504 primary HNC [12]. The 1-year, 1.5-year, and 2-year cumulative incidence of a secondary event local recurrence (LR), regional recurrence (RR), second primary head and neck cancer (SPHNC) were 10, 12, and 13% for oral cavity cancer (OCC); 6, 10, and 11% for oropharyngeal cancer (OPC); 7, 11, and 13% for laryngeal cancer and 11, 19, and 19% for hypopharyngeal cancer (HPC). Most recurrences, almost independent of the primary site, occurred during the first 1–2 years. The authors calculated pick-up rates (one detected recurrence/number of visits) for various sites according to detected recurrences during follow-up time frame and number of expected guideline-prescribed follow-up visits. For OCC pick-up rate after 2 years of follow-up was 1/136. For OPC, LC, and HPC after 1.5 follow-up years led to a pick-up rate of 1 in 235, 1 in 144, and 1 in 237 visits, respectively. The authors concluded that only 1 year of follow-up for OCC, and 1.5 years for OPC, LC, and HPC, suffices for the goal of detecting disease manifestations after treatment [12].

Recent detailed analysis of altogether 447 patients diagnosed with recurrent HNC gave similar numbers. In this cohort, 50.6% of recurrences occurred within 6 months of treatment completion, 72.5% occurred within 1 year, and 88.6% occurred within 2 years [13].

Most previous studies conclude that follow-up for OCC may be inefficient after the first 3 years of follow-up [14]. In a retrospective study of 594 OCC patients, almost all locoregional recurrences occurred in the first 2 years after treatment. The incidence of second primary tumours was relatively stable over the years. The 1-year, 2-year and 3-year cumulative risks of all recurrences were 17, 30, and 37%. The authors concluded that 2 years follow-up time would be sufficient for oral cancer [14]. Similar results were obtained by Blatt *et al.*, who evaluated clinical records of 831 consecutive patients diagnosed with primary OCC. A total of 216 (28%) patients had recurrence and of those only 24% occurred after 12 months. Of all detected recurrences, 27% were primarily diagnosed with CT imaging [15].

Low recurrence rate of OPC after 2–3 years was noticed in the national study from Finland where 495 OPC patients were evaluated. Detected number of locoregional recurrences, analysed by population at risk, during first 2, 2–3, and 3–5 years after treatment was 23, 7, and 3% respectively. There was a significant difference in recurrence rate between early and advanced T stage during first 2 years after treatment (6 vs. 17%). Of all recurrences, 86% were diagnosed between 6 and 36 months after primary treatment [16]. Recent two series focusing on early-stage OPC showed also low recurrence rates during surveillance. One-year recurrence free survival (RFS) was 96% and 3 years RFS 87.8–98.6% [17^▪^,18^▪^], which agrees other recent study including also advanced stage diseases showing cumulative incidence of all recurrences of 12.6% during 4.5 years FU [19].

Low incidence of recurrences was also shown in a cohort study of 888 OPC patients. The 2-year and the 5-year cumulative rates of disease recurrence were 2.6 and 5.6%, respectively, in the p16-positive patients, and they were 14.1%, and 20.5% in the p16-negative patients, respectively. The time from the end of the initial treatment to the first disease recurrence was similar between the p16-positive and in the p16-negative patients. The distributions of LRR alone, isolated distant metastasis (DM) recurrence, or concurrent locoregional recurrence (LRR) and DM recurrence were 41.8, 37.2, and 20.9%, respectively, in the p16-positive patients, and 60.4, 22.8, and 16.8% in the p16-negative patients (*P* = 0.13) [20].

### Second primary cancer

Detection of secondary primary cancer (SPC) has been regarded as an important rationale for HNC surveillance, as its incidence has been shown to be higher in HNC patients than in normal population. Risk of developing an SPC rises exponentially over time, with SPM risks of 4, 10, and 25% at 5, 10, and 15 years, respectively [21].

The elevated and persistent, risk of SPC is supported by Finnish Cancer Registry study, which showed an 85% excess risk among patients with OCC, and the incidence remained stable for over 20 years (standardized incidence ratio 1.65–2.05) [22]. The similar increased and stable risk of SPC was shown also among patients with laryngeal cancer (SIR 1.53–1.73 1–20 years after treatment) [23]. Also, van de Weerd *et al.* [24^▪▪^] showed that the incidence of SPC does not decrease beyond 5 years of follow-up. In the national study of 2584 patients from Danmark, median time to SPC after a primary OPC was 2 years and human papillomavirus (HPV)-positive patients had a significantly longer median time to SPC than HPV-negative patients. Patients with primary OCC had a relatively low risk of a SPC, while patients with an index tumour of the hypopharynx had the highest risk [25].

### Value of scheduled follow-up vs. patient-driven follow-up

Timing of recurrence and survival outcomes according to the modality of detection of recurrence was evaluated in the series of 326 patients with stage III–IV HNC from various sites. A locoregional radiologic evaluation was performed at least twice per year for the first 2 years and yearly in the following 2 years. Recurrence or a second primary tumour was diagnosed with 106 patients (32%). Eighty-four percent (32 of 38) were diagnosed in the first 3 years of follow-up. Approximately 70% of recurrences were clinically and/or radiologically discovered, whereas 30% were diagnosed due to the patients’ symptoms. The observed detection rate – defined as the number of ENT visits performed to detect relapse or SPC – was 0.5% (1 of 200 visits). Even though most of the recurrences were diagnosed in scheduled follow-up visits by clinical examination or radiological imaging, overall survival did not differ according to the type of detection modality [26].

Limited benefit of routine control visits on survival was seen in a retrospective study of 413 LC patients. For patients with early-stage LC diagnosed with LRR at a routine-driven or patient-driven visit, the 5-year OS was similar: 70 and 72%, respectively. For patients with advanced-stage LC, overall survival (OS) was better if recurrence was diagnosed at a routine visit, the 5-year OS was 37% compared to 18% for detection at a patient-driven visit. However, after advanced-stage LC, no asymptomatic recurrences were detected beyond 1 year posttreatment despite regular follow-up. Authors concluded that routine follow-up for detecting asymptomatic recurrences seems redundant for early-stage LC [24^▪▪^].

Critical opinion regarding prolonged follow-up was presented in the study of 456 patients with HNC. A total of 94 (22%) patients relapsed during the 5-year follow-up period, and 90% of all recurrences were found within 3 years. All recurrent tumours found during routine follow-up visits without symptoms were found within 34 months after completion of treatment [27].

Rate of asymptomatic recurrences in OPC patients was studied by analysing all the consecutive FU visits during one year period in one institution. During one year period, four recurrences were detected during 366 follow-up visits in 153 patients and. In total, 340 of 366 (93%) outpatient visits were from patients presenting without new symptoms, and not a single recurrence was found during these visits [28]. In another study focusing on hypopharyngeal cancer, 51 out of 60 recurrences developed new symptom before diagnosis of recurrence [29^▪▪^].

### Value of imaging in head and neck cancer surveillance

MD Anderson Cancer Center assessed the efficacy of surveillance imaging in a large series of 1508 patients with HNC [30]. Of recurrences, 75 were clinical-detected and 58 were imaging-detected. The authors concluded that the majority of recurrences occurred within the first 2 years posttreatment with 47% detected via imaging alone, and it is reasonable to perform surveillance imaging within this follow-up time period [30].

Value of imaging in early detection of recurrence was studied in a single centre study of 255 HNC patients with recurrences. With scheduled imaging of neck CT every 6 months up to 2 years, proven recurrences were detected by imaging alone in 59 of 255 patients, and the authors concluded that imaging should be extended beyond 6 months [31^▪^]. Similar figures were also shown by Farsi *et al*. who retrospectively evaluated 72 recurrent HNC patients. The method of initial detection spurring further investigation was imaging in 42 (58.3%) patients and clinical examination in 30 (41.3%) patients [32^▪^].

Utility of surveillance imaging within 2 years from treatment was also seen in study of 276 HPV-associated OPC patients. Of all recurrences, 11 (39.3%) were detected by the first posttreatment scan, 11 (39.3%) by surveillance scan and 5 (17.9%) by clinical exam. The number of surveillance scans needed to detect one recurrence was 45 within 2 years, and 248 beyond 2 years from treatment [33].

Retrospective study of 340 patients, focusing on oncologic outcomes, failed to show efficacy of surveillance imaging in asymptomatic patients. A total of 187 patients (55%) underwent imaging-based surveillance (CT, MRI, or PET-CT), and 153 (45%) were treated expectantly with imaging reserved only in the presence of suspicious symptoms and/or physical findings. There was no difference in 3-year local-regional control, overall survival, progression-free survival, or freedom from distant metastasis between patients treated with surveillance imaging vs. those treated expectantly [34].

The association of posttreatment follow-up imaging with survival was found by Leclere *et al.* Patients undergoing an intensive posttreatment follow-up strategy had PET/CT at months 12, 24, and 36, chosen at the discretion of head and neck surgeons, and this group was compared to conventional follow-up (CFU). Four hundred and ninety-seven patients had 18FDG-PET/CT during follow-up and 285 patients had CFU. The mean (SD) 3-year OS was significantly better in the PET/CT vs. CFU group (72.5 vs. 64.3%) [35]. Small benefit of imaging in improving survival was found in the population-based study with 1004 patients. Surveillance imaging was associated with lower mortality among patients with HNC with regional or distant disease, and this was observed 2 years after treatment. However, as a registrar study, some inherent risk of biases may exist [36].

Comprehensive review by Van Hoe and Hermans supports surveillance imaging at least for one or probably 2 years after treatment. They selected 48 articles for the final review, of which CT and PET-CT were mostly the imaging techniques. A large majority (18/25) of reports analysed in this study suggest that systematic posttreatment surveillance imaging may be useful in terms of additional lesion detection when compared to a strategy where imaging is reserved for cases with suspect symptoms or clinical findings. Most authors in their review agreed that imaging screening during 1–2 years may be effective but after 2 years its value was left uncertain [37^▪▪^].

## CONCLUSION

Most accepted surveillance programs have been generated decades ago and since then stated as irreplaceable. Currently, many other specialists in addition to head and neck surgeons and oncologists have been involved in multidisciplinary pretreatment and surveillance programs, such as cancer-specialist nurses, psychologists, speech-therapists, physiotherapists, and social workers. The recent literature has provoked discussion about separating the follow-up programs focusing only on early detection of possible recurrences from other important aspects of surveillance, by utilizing all those other specialists to rehabilitate and support patients when needed [1,38^▪^].

Results of the studies focusing on the efficacy of routine follow-up visits (including value of imaging) vs. patient-driven symptomatic visits are strongly affected by the study population, recurrence rate, intensity of follow-up programs, patient's accessibility to treatment centre and imaging-specific factors. However, according to recent studies 70–90% of locoregional recurrences and most of the distant metastases seems to occur during the first 1–2 years after treatment, depending on the primary site and stage of the disease.

Screening of SPC after HNC may be justified, but according to recent studies, screening for this indication should not be terminated even after 5 years, because the incidence of SP remains stable or even increases at least 10–20 years after primary HNC. One trial demonstrated lung cancer screening for individuals aged 50–79 with at least 20 pack-years, showing a 20% improvement in lung cancer mortality with annual low-dose CT chest imaging, translating to a 6.7% increase in OS [39].

The value of imaging in HNC surveillance in terms of survival has not been fully confirmed. Patients, however, do find imaging indispensable to diminish their fear and concerns about recurrences. Further, some evidence supports imaging at least 1–2 years after treatment, which improves possibilities for earlier recurrence detection, at least for high-risk patients. Focusing on recurrences, which possibly would be salvageable, the most efficient time -period could be 2 years, and in some cases, 3 years. After 2–3 years from treatment, incidence of recurrences significantly decreases, and the pick-up rate drops to a considerably low 1/150–1/250 visits. Terminating routine follow-up at 2–3 years would evidently mean that sporadic recurrences would be detected only by the patient's subjective symptoms. However, almost all late recurrences are symptomatic [27]. Comprehensive patient education and easy access to treatment centre due to alarming symptoms would probably still increase the patient-referred sensitivity in detecting late recurrences.

Some recent studies bring up benefits of personalized and risk-related surveillance programs, but as stated, strong evidence for stratification patients into various risk groups according their stage of the disease or risk factors does not exist [40^▪^,^41^,^42^]. In the absence of randomized prospective evidence, our decision-making depends on retrospective data analyses and expert opinion. According to recent studies, routine follow-up protocols at least in high-risk patients should continue 2–3 years after treatment, but long-term surveillance in terms of detecting asymptomatic recurrences may be ineffective. Most studies support also routine imaging for at least 1–2 years after treatment, but its impact on survival outcomes has not been established.

## Acknowledgements

*None.*

### Financial support and sponsorship

*None.*

### Conflicts of interest

*There are no conflicts of interest.*

## REFERENCES AND RECOMMENDED READING

Papers of particular interest, published within the annual period of review, have been highlighted as:

▪ of special interest
▪▪ of outstanding interest
